# An oxidative stress-related signature for predicting the prognosis of liver cancer

**DOI:** 10.3389/fgene.2022.975211

**Published:** 2023-01-04

**Authors:** Luling Wang, Xing Liu

**Affiliations:** Department of Central Laboratory Medicine, Shanghai Municipal Hospital of Traditional Chinese Medicine, Shanghai University of Traditional Chinese Medicine, Shanghai, China

**Keywords:** liver cancer, oxidative stress, prognostic signature, nomogram, immune microenviroment

## Abstract

**Introduction:** This study aimed to screen for oxidative stress-related genes (OSRGs) and build an oxidative stress-related signature to predict the prognosis of liver cancer.

**Methods:** OSRGs with a protein domain correlation score ≥ 6 were downloaded from the GeneCards database and intersected with The Cancer Genome Atlas (TCGA) data for subsequent analyses. Differential immune cells (DICs) and immune and stromal scores between the normal and tumor samples were determined, followed by unsupervised hierarchical cluster analysis. Immune-related OSRGs were identified using weighted gene co-expression network analysis. An OSRG-related risk signature was then built, and the GSE14520 dataset was used for validation. A nomogram evaluation model was used to predict prognosis.

**Results:** Nine DICs were determined between the normal and tumor groups, and three subtypes were obtained: clusters 1, 2, and 3. Cluster 1 had the best prognosis among the clusters. One hundred thirty-eight immune-related OSRGs were identified, and seven prognosis-related OSRGs were used to build the OSRG score prognostic model. Patients in the high OSRG score group had a poorer prognosis than those in the low OSRG score group. Six immune cell infiltration and enrichment scores of the 16 immune gene sets showed significant differences between the high and low OSRG score groups. Moreover, a nomogram was constructed based on the prognostic signature and clinicopathological features and had a robust predictive performance and high accuracy.

**Conclusion:** The OSRG-related risk signature and the prognostic nomogram accurately predicted patient survival.

## 1 Introduction

Liver cancer is the most critical cancer and tumor disease, threatening the safety of human life and is a common malignant tumor with high incidence and mortality (Marengo et al., 2016). With the increase in social work pressure and changes in living habits, the incidence of liver cancer has increased annually. In 2020, 905,677 new cases and 830,180 deaths from liver cancer were reported worldwide (Sung et al., 2021), with 41,438 new cases and 391,152 deaths in China, accounting for 47.1% of all liver cancer-related deaths worldwide (Cao et al., 2021). Presently, the pathogenesis and mechanism of liver cancer have not been fully elucidated. However, hepatitis B virus infection, immune response, and inflammatory factor response are the leading causes of liver cancer (Jin et al., 2019). Surgery, radiotherapy, and chemotherapy are the primary treatment methods, but recurrence and metastasis of liver cancer seriously affect the therapeutic effect, prognosis, and survival of patients (Boland and Wu, 2018). A comprehensive study of the molecular mechanisms of liver cancer and identifying molecular targets are of great significance for the prognosis and targeted therapy of liver cancer.

Oxidative stress is an imbalance between detoxification and reactive oxygen species (ROS) production in the body (Sajadimajd and Khazaei, 2018). Free radicals negatively affect the body and are considered vital factors contributing to disease and aging (Kudryavtseva et al., 2016). DNA damage and incorrect repair caused by oxidative stress can contribute to the activation of oncogenes or inactivation of tumor suppressor genes, which can induce cancer. Oxidative stress can also facilitate tumor neovascularization and enhance the growth and metastasis of cancer cells (Klaunig, 2018; Sajadimajd and Khazaei, 2018). Using antioxidants to capture free radicals and eliminate or reduce oxidative stress is a valuable method to prevent cancer development (Chikara et al., 2018). Studies have found that people who consume antioxidant-rich fruits and vegetables have a relatively low incidence of cancer (Lin et al., 2019; Kim et al., 2020). Thus, there is an urgent need to identify biomarkers to predict the response to oxidative therapy for liver cancer.

Growing evidence suggests that changes in the tumor immune microenvironment are associated with tumor survival and progression (Soysal et al., 2015; Hinshaw and Shevde, 2019). However, mechanisms underlying oxidative stress and its association with the immune microenvironment in liver cancer remain unclear. This study aimed to screen immune-associated oxidative stress-related genes (OSRGs) and build an oxidative stress-related signature to predict liver cancer prognosis. This study provides insights into immune biomarkers and therapeutic strategies for liver cancer treatment.

## 2 Materials and methods

### 2.1 Data collection

Gene expression levels [normalized log_2_ (FPKM+1) expression level data] and clinical follow-up data of 50 normal and 371 liver cancer samples were obtained from The Cancer Genome Atlas (TCGA) database. Samples with missing overall survival (OS) times and 0 were removed, and 365 liver cancer samples with prognostic information were retained. In addition, the GSE14520 dataset (Wang C. et al., 2021) with prognosis information for liver cancer was acquired from the Gene Expression Omnibus (GEO) database (Barrett et al., 2013), and 242 liver cancer samples were retained for subsequent validation analysis after excluding the samples with missing OS time and 0.

### 2.2 Acquisition of OSRGs

OSRGs with a protein domain correlation score ≥6 (minimum standard value) were downloaded from the GeneCards database and intersected with TCGA data for subsequent analyses.

### 2.3 Immune microenvironment in normal and tumor samples

CIBERSORT (Chen et al., 2018) was used to evaluate the proportion of 22 immune cells according to their expression levels in TCGA‒liver hepatocellular carcinoma samples. In addition, the R package ‘estimate’ (Yoshihara et al., 2013) was used to evaluate immune and stromal scores. The Wilcoxon test was used to compare the differential immune cells (DICs), immune scores, and stromal scores.

### 2.4 Unsupervised hierarchical cluster analysis

Based on the infiltration score of the obtained DICs, unsupervised hierarchical cluster analysis was conducted using ConsensusClusterPlus (Wilkerson and Hayes, 2010) to predict the subtypes of patients with liver cancer, and the optimal tumor subtype (K value) was obtained with a threshold of K = 2–6. Kaplan‒Meier analysis was employed to assess the survival differences between patients with different subtypes using the survival package (Rizvi et al., 2019). The correlations between prognostic subtypes and clinical information (age, sex, grade, pathological T/N/M, and stage) were evaluated using the chi-squared test based on integrated clinical information data of liver cancer.

### 2.5 Weighted gene co-expression network analysis (WGCNA)

WGCNA in R (Langfelder and Horvath, 2008) was utilized to identify immune-related gene modules with the characteristics of immune subtype and immune and stromal scores, and the threshold was set to *p* < 0.05. Immune-related OSRGs have also been identified.

### 2.6 Construction and validation of a risk model

Univariate Cox regression analysis was performed on immune-related OSRGs using the ‘survival’ package to screen prognosis-related OSRGs with a cutoff value of *p* < 0.05. The key OSRGs were obtained using least absolute shrinkage and selection operator (LASSO) regression with 10-fold cross-validation and a *p*-value <0.05. Stepwise Cox regression analysis was used to build the OSRG-related risk model using the ‘survminer’ package. The OSRG score was calculated using the following formula: OSRG score = h_0_(t) * exp (*β*_1_X_1_ + β_2_X_2_ + ... +β_n_X_n_) [the *β* gene represents the regression coefficient, h_0_(t) represents the benchmark risk function, X_n_ indicates the concomitant variable, and h (t,X) indicates the risk function associated with X at time t]. The samples in TCGA and GSE14520 datasets were categorized into high- and low-OSRG score groups according to the median cutoff value of the OSRG score. Kaplan‒Meier analysis was employed to assess the difference in survival between patients in the high- and low-OSRG score groups. In addition, the correlations between high and low OSRG scores and clinical information (age, sex, grade, pathological T/N/M ratio, and stage) were evaluated using the chi-squared test.

### 2.7 Immune microenvironment

The microenvironment cell population counter (MCPcounter) algorithm was employed to assess the immune cell infiltration score, and the GSVA algorithm (Shi et al., 2020) was used to calculate the enrichment score of 16 immune gene sets obtained from the Immport database (Bhattacharya et al., 2014). The Wilcoxon test was used to compare the immune cell infiltration and enrichment scores of the 16 immune gene sets between the high- and low-OSRG score groups.

### 2.8 Gene set enrichment analysis (GSEA)

Based on the expression profile in patients with liver cancer, GSEA (Reimand et al., 2019) was performed to compare the differences in the HALLMARK gene set between the high- and low-OSRG score groups with cutoff values of *p* < 0.05 and normalized enrichment score (NES) > 1.

### 2.9 Nomogram construction

Univariate and multivariate Cox regression analyses were conducted to investigate the relationships between clinical factors and the OSRG score to screen for independent prognostic factors with a cutoff value of *p* < 0.05. Additionally, the screened independent prognostic factors and OSRG scores were used to build a nomogram with the “rms” package (Zhang et al., 2019) in R. The performance of the nomogram was validated by measuring the concordance index (C-index), calibration curves, and receiver operating characteristic (ROC) curve.

## 3 Results

### 3.1 Immune microenvironment between normal and tumor samples

**FIGURE 1:**
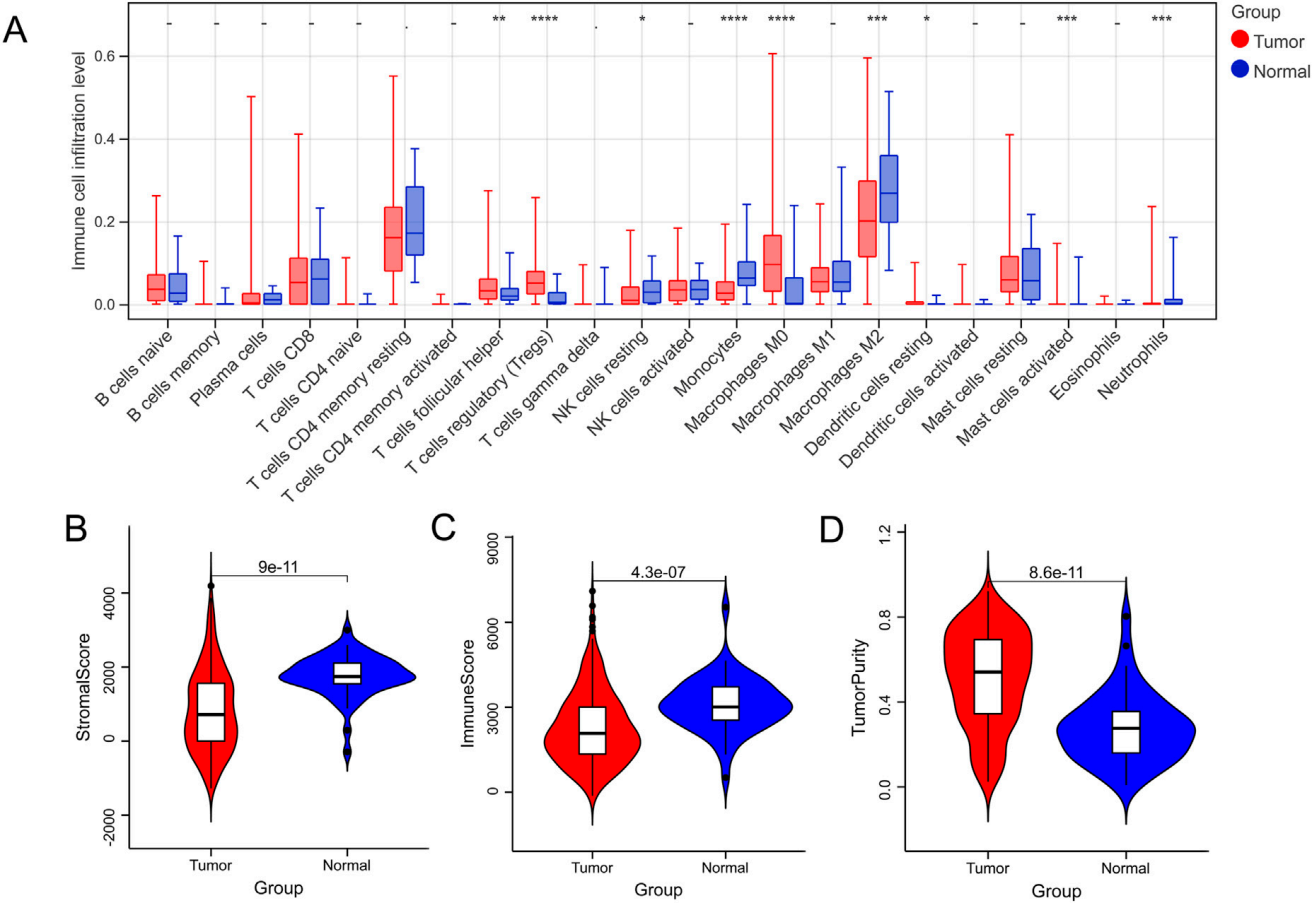
Immune microenvironment between normal and tumor samples. **(A)** The difference in immune cells between normal and tumor groups. **(B–D)** Tumor microenvironment scores between normal and tumor groups. *p < 0.05, **p < 0.01, ***p < 0.001, –*P* > 0.05.

In total, nine DICs were obtained between the normal and tumor groups (Figure 1A), and the tumor group had the lowest immune and stromal scores and the highest score of tumor purity (Figures 1B‒D).

### 3.2 Identification of three immune subtypes

**FIGURE 2:**
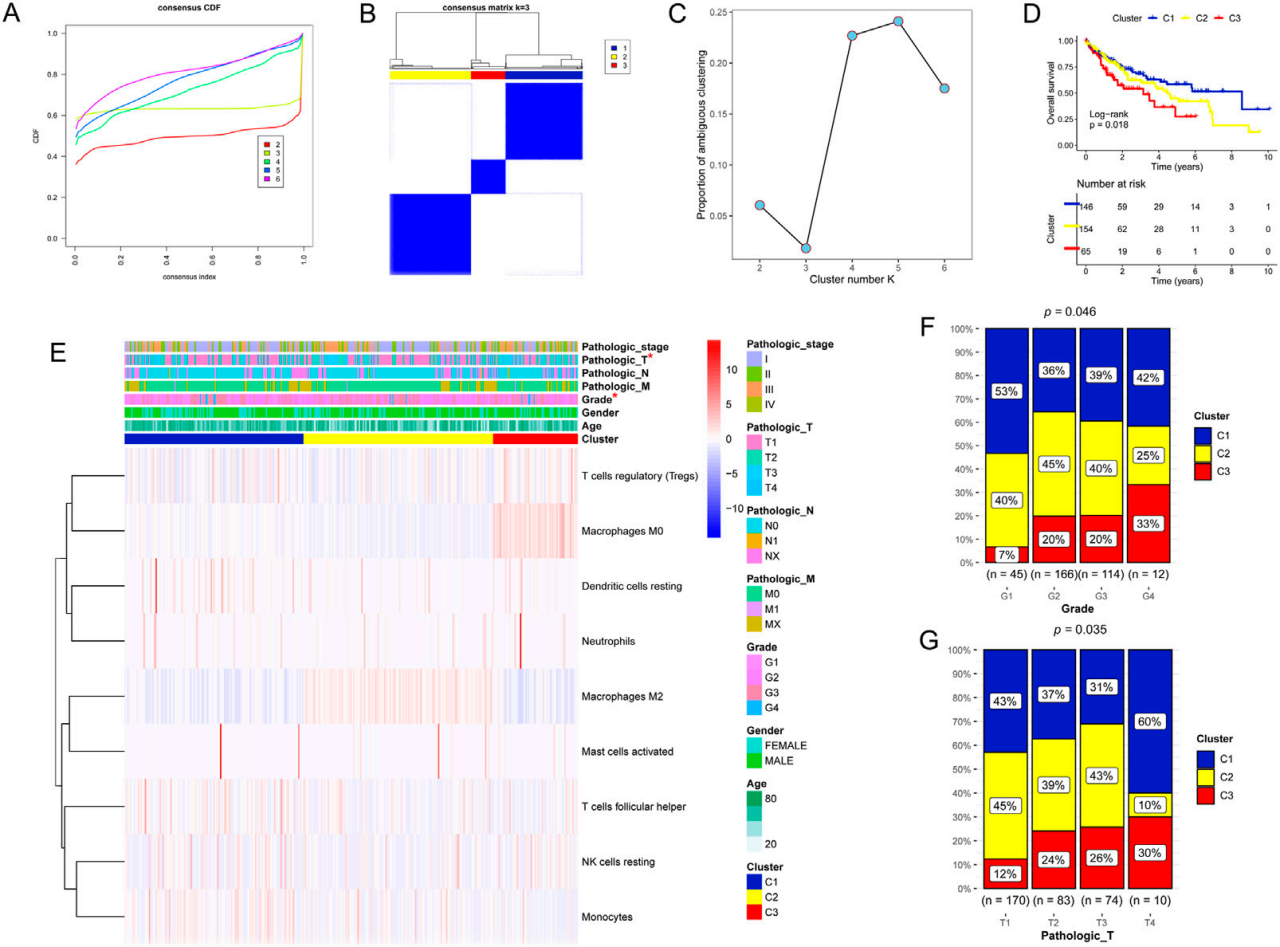
Unsupervised hierarchical cluster analysis. **(A–C)** Three molecular subtypes were identified. **(D)** Kaplan‒Meier analysis of the three molecular subtypes. **(E–G)** Proportion of clinicopathologic features of the three molecular subtypes.

Based on the nine DICs, unsupervised hierarchical cluster analysis was performed, and the optimal tumor subtype was K = 3 (Figure 2A). Three subtypes were obtained, namely clusters 1, 2, and 3, with 146, 154, and 65 liver cancer samples, respectively (Figure 2B). Proportion of ambiguous clustering (PAC) analysis further verified that the optimal tumor subtype was *K* = 3 (Figure 2C). Survival analysis showed that cluster 1 had the best prognosis among all the clusters (Figure 2D). The correlations between the prognostic subtypes and clinical information are shown in Figures 2E‒G.

### 3.3 Immune-related OSRGs

**FIGURE 3:**
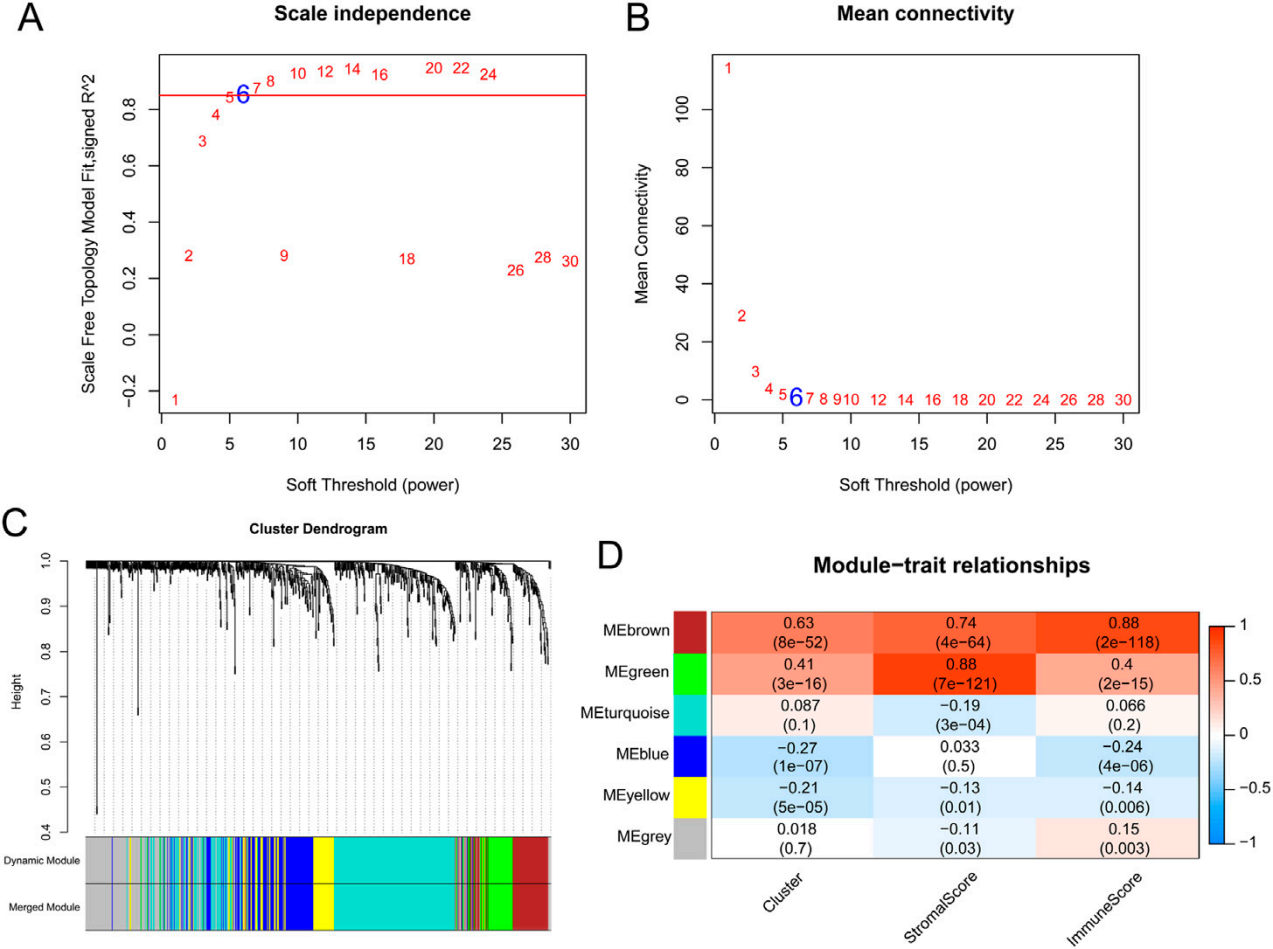
The results of weighted gene co-expression network analysis. **(A)** Estimation of the scale independence index of the 1–30 soft threshold power (*β* = 6). **(B)** Determination of the mean connectivity of the 1–30 soft threshold power. **(C)** Tree diagram for module division (different color represent different modules). **(D)** Relationships of the module with the cluster and immune and stromal scores.

Based on the 758 OSRGs, the soft threshold power for matrix transformation was determined to be 6, where the square of the correlation coefficient between log2k and log2p k) was 0.85 (Figures 3A, B). Six modules were screened, and the correlation between clinical features and each module was calculated. The brown and green modules, which had the highest correlation with clinical features, were selected as the key modules and had 80 and 58 immune-related OSRGs, respectively (Figures 3C, D).

### 3.4 OSRG score prognostic model

**FIGURE 4:**
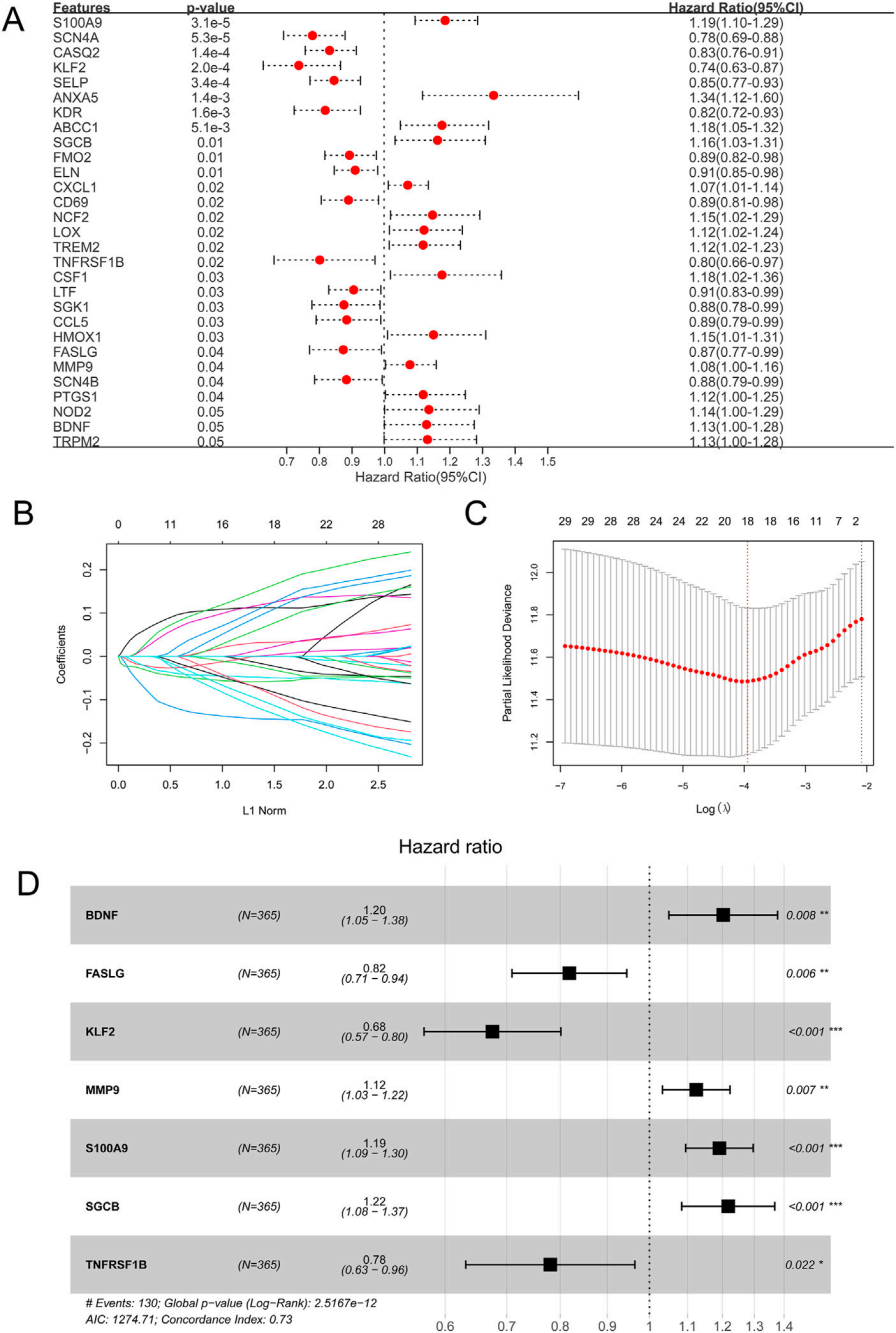
Oxidative stress-related gene (OSRG) score prognostic signature. **(A)** Univariate analysis of immune-related OSRGs. **(B)** Least absolute shrinkage and selection operator (LASSO) coefficient profiles of the 29 survival-related genes. **(C)** Coefficient profile plot produced against the log(lambda) sequence in the LASSO model. The optimal parameter (lambda) was selected as the first black dotted line indicated. **(D)** Stepwise Cox regression analysis. *p < 0.05, **p < 0.01, ***p < 0.001.

**FIGURE 5:**
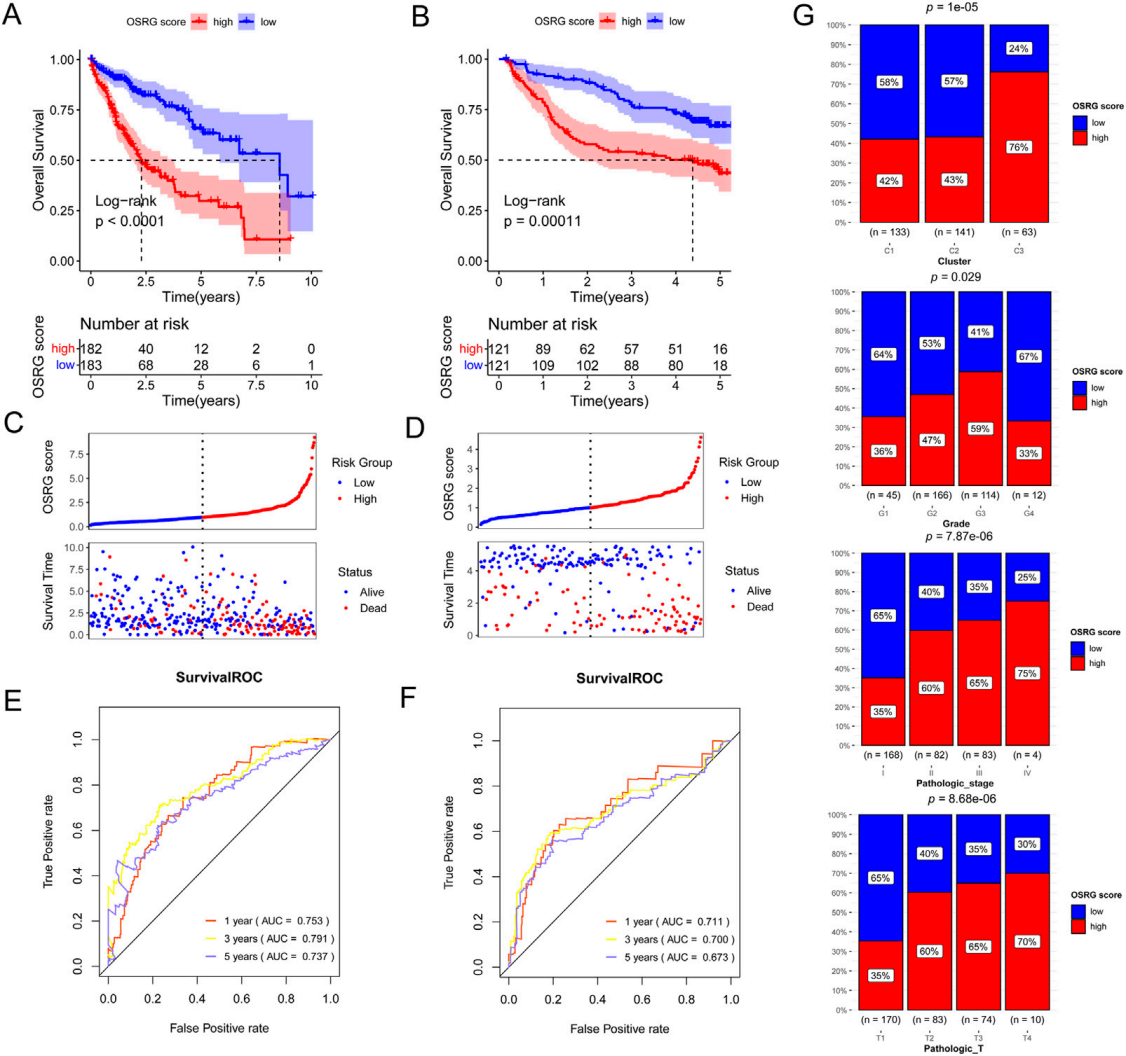
Generation, evaluation and validation of an OSRG score prognostic signature. Kaplan-Meier analysis of the low and high OSRG score groups in The Cancer Genome Atlas (TCGA) cohort **(A)** and GSE14520 dataset **(B)**. Risk score distribution and survival status in TCGA cohort **(C)** and GSE14520 dataset **(D)**. The areas under the receiver operating characteristic curves (AUCs) for predicting 1-, 3-, and 5-year overall survival (OS) in TCGA cohort **(E)** and GSE14520 dataset **(F)**. **(G)** the correlations between high and low OSRG scores and clinical information.

Based on 138 immune-related OSRGs, 29 prognosis-related OSRGs were obtained after univariate Cox regression analysis (Figure 4A). LASSO regression analysis was conducted on the 29 prognosis-related OSRGs, resulting in 18 OSRGs (Figures 4B, C). Stepwise Cox regression analysis resulted in seven OSRGs, *BDNF*, *FASLG*, *KLF2*, *MMP9*, *S100A9*, *SGCB*, and *TNFRSF1B*, which were used to build the OSRG score prognostic model (Figure 4D). Patients in the high-OSRG score group had a poorer prognosis than those in the low-OSRG score group (*p* < 0.05; Figures 5A,B). The distribution of the OSRG score and survival status in TCGA and GSE14520 datasets are shown in Figures 5C, D, respectively. The areas under the curve (AUCs) at 1, 3, and 5 years for OS in the TCGA dataset were 0.753, 0.791, and 0.737, respectively, and in the GSE14520 dataset were 0.711, 0.700, and 0.673, respectively (Figures 5E,F). In addition, the correlations between high and low OSRG scores and clinical information (age, sex, grade, pathological T/N/M, and stage) were evaluated, and the results showed that OSRG scores were significantly related to clusters, grade, pathological T, and stage (Figure 5G).

### 3.5 Immune microenvironment between the high and low OSRG score groups

**FIGURE 6:**
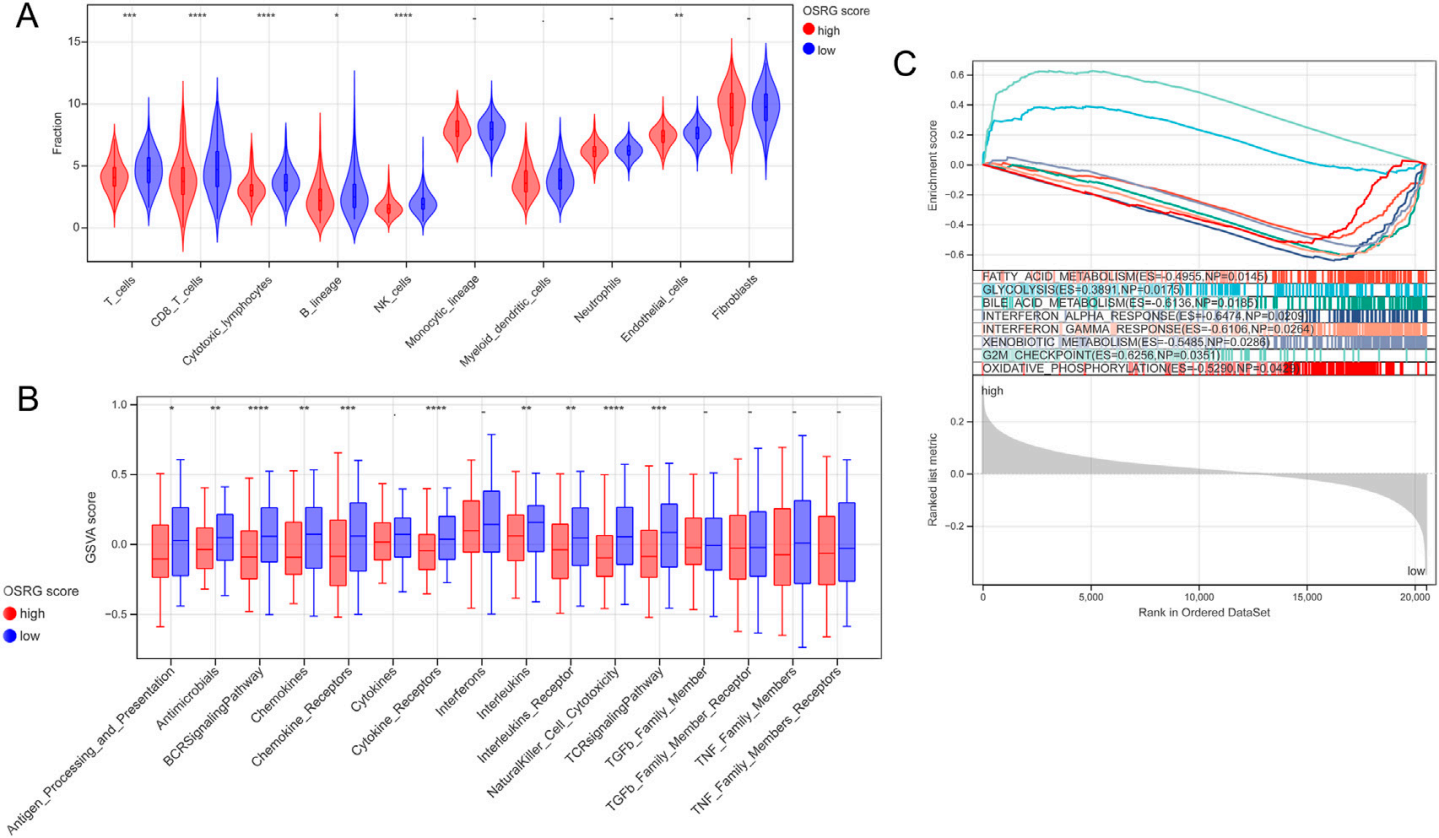
Immune microenvironment and gene set enrichment analysis (GSEA) analysis between the high and low OSRG score groups. **(A)** Six differential immune cells were obtained between the high and low OSRG score groups. **(B)** The enrichment score of 16 immune gene sets showed significant differences between the high and low OSRG score groups. **(C)** Eight HALLMARK gene set enrichment pathways were significantly different between the high- and low-OSRG score groups. *p < 0.05, **p < 0.01, ***p < 0.001, -*P* > 0.05.

The MCPcounter algorithm was used to evaluate the immune cell infiltration score according to the expression profile data of TCGA samples, and the results showed six DICs between the high- and low-OSRG score groups (Figure 6A). The enrichment scores of the 16 immune gene sets showed significant differences between the high- and low-OSRG score groups (Figure 6B).

### 3.6 GSEA

GSEA was performed to compare the differences in the HALLMARK gene set between the high- and low-OSRG score groups with a cutoff value of *p* < 0.05 and NES >1. The results showed that the enrichment pathways of the eight HALLMARK genes were significantly different between the two groups (Figure 6C), including fatty acid metabolism, glycolysis, and bile acid metabolism.

### 3.7 Construction of a nomogram for predicting patient OS

**FIGURE 7:**
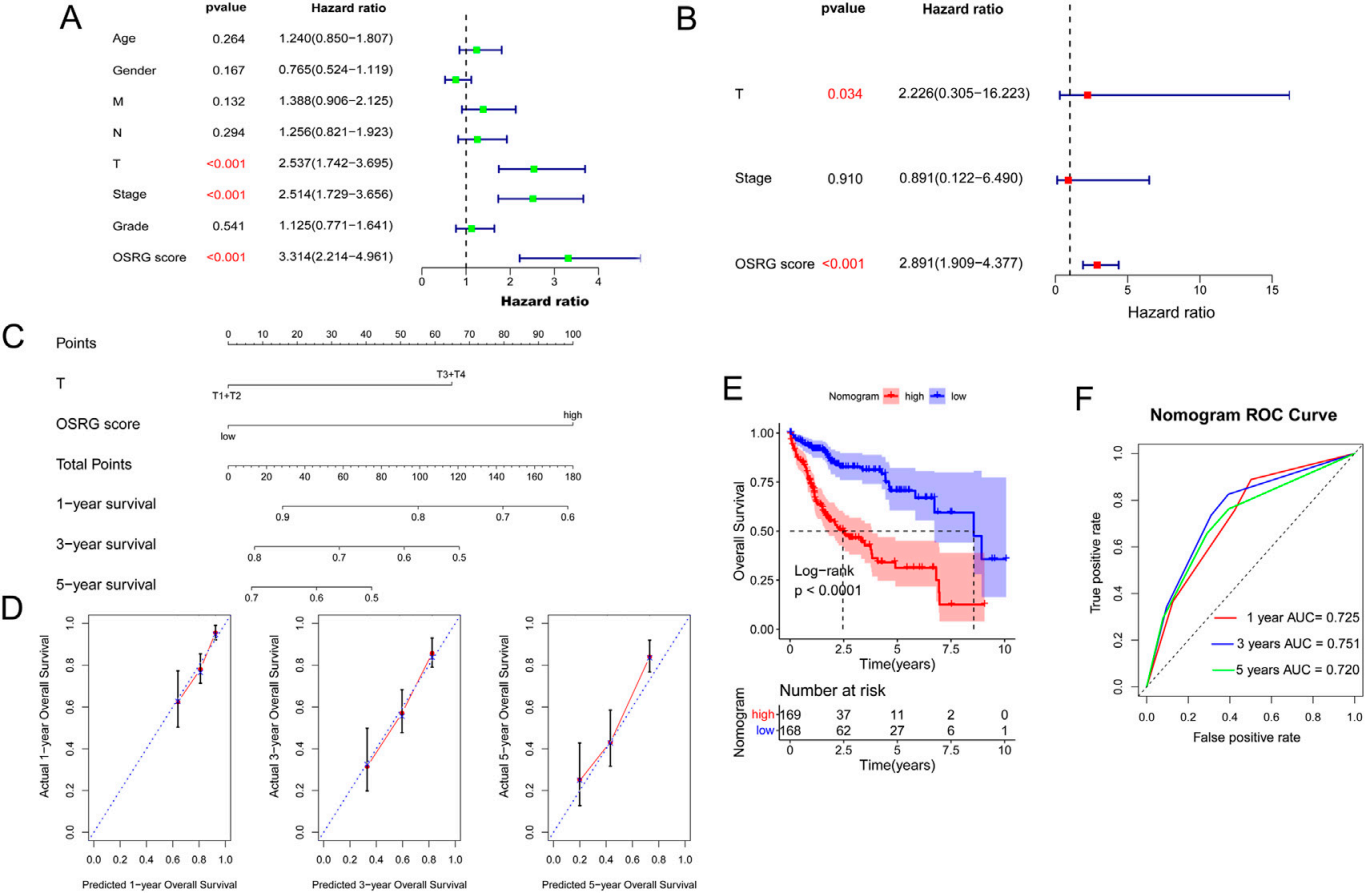
Construction of a nomogram. **(A)** Univariate analysis of OSRG score and clinicopathological characteristics. **(B)** Multivariate analysis of OSRG score and clinicopathological characteristics. **(C)** A nomogram for predicting 1-, 3-, and 5-year OS. **(D)** Calibration curves for predicting 1-, 3-, and 5-year OS. **(E)** Kaplan‒Meier analysis of the nomogram. **(F)** The AUCs for predicting 1-, 3-, and 5-year OS.

To explore the relationship between clinicopathological features and the prognostic model, age, sex, pathological MNT, stage, grade, and OSRG score in TCGA samples were analyzed, which revealed that pathological T and OSRG scores were independent prognostic factors (*p* < 0.05; Figures 7A, B), and the C-indices of the pathological T and OSRG scores were 0.600 and 0.687, respectively. Moreover, a nomogram was built with pathological T and OSRG scores (Figure 7C), and its performance was validated by measuring the C-index, calibration curves, and ROC curve. The results showed that the C-index of the nomogram was 0.703, and calibration plots revealed that the nomogram accurately estimated mortality (Figure 7D). Survival analysis revealed that the nomogram was significantly associated with the patient’s prognosis (Figure 7E). The nomogram ROC curves showed that the AUCs at 1, 3, and 5 years were 0.725, 0.751, and 0.720, respectively (Figure 7F). These findings suggest the appreciable reliability of the nomogram.

## 4 Discussion

Liver cancer is a common malignant tumor worldwide (Sia et al., 2017). Oxidative stress significantly affects various functions and processes, such as cell proliferation, differentiation, angiogenesis, and metabolism, and is related to the pathophysiology of various diseases (Klaunig, 2018). Thus, this study aimed to screen for OSRGs and build an oxidative stress-related signature to predict liver cancer prognosis. Nine DICs were found between the normal and tumor groups, including regulatory T cells (Tregs), monocytes, and M0 macrophages. Tregs are a subset of T cells specifically used to control immune responses and have been shown to play a vital role in regulating autoimmune diseases, cancers, and infectious diseases (Sakaguchi et al., 2020). Wang et al. (2014) found that methionine enkephalin improved peripheral blood lymphocyte subsets by inhibiting Tregs in 50 cancer patients. Monocytes are innate immune cells of the monocyte phagocyte system that have become an important regulatory factor in tumor development (Olingy et al., 2019). Tumor-associated macrophages are vital in promoting tumor progression (Pucci et al., 2021). Therefore, these nine DICs may play an important role in liver cancer progression. In addition, unsupervised hierarchical cluster analysis was performed based on the nine DICs, and three subtypes were obtained: clusters 1, 2, and 3. Survival analysis showed that cluster 1 had the best prognosis among the clusters, and cluster 1 had a lower grade and pathological T, according to the survival analysis results.

Seven OSRGs were included in the OSRG score prognostic model: *BDNF*, *FASLG*, *KLF2*, *MMP9*, *S100A9*, *SGCB*, and *TNFRSF1B*. Brain-derived neurotrophic factor (BDNF) is a potent neurotrophic factor (Numakawa et al., 2018) that promotes breast cancer cell growth and metastasis (Tajbakhsh et al., 2017). Oyama et al. revealed that tropomycin receptor kinase B (TrkB)/BDNF signaling could be a novel therapeutic target in pancreatic cancer (Oyama et al., 2021). Liu et al. (2009) found that Fas ligand (FASLG) polymorphisms were correlated with cancer risk. In a study by Wang G. et al. (2021), lncRNA CASC7 inhibited breast cancer malignant behaviors by regulating the miR-21-5p/FASLG axis. Gao et al. (2019) showed that Kruppel-like factor 2 (KLF2) prevents osteoarthritis *in vitro* and *in vivo* by activating the Nrf2/ARE signaling pathway to inhibit oxidative responses. Lu et al. (2021) revealed that in clear-cell renal cell carcinoma (RCC), KLF2 regulated ferroptosis through GPX4, thereby inhibiting cell migration and invasion. Matrix metallopeptidase-9 (MMP-9) has been widely associated with cancer pathology (Huang, 2018). *S100A9* is an oxidative stress gene (Saheb Sharif-Askari et al., 2021), and Lv et al. (2020) found that S100A9 enhances prostate cancer cell invasion by activating TLR4/NF-κB/integrin β1/FAK signaling. Chen et al. (2021) found that β-sarcoglycan (*SGCB*) is a prognostic metabolism-related gene in clear-cell RCC. Yu et al. (2014) found that mutations in the inflammation-related gene *TNFRSF1B* might alter the risk of colorectal cancer. These seven OSRGs were used to build an OSRG score prognostic model. Patients in the high OSRG score group had a poorer prognosis than those in the low OSRG score group. Regarding OS, the AUCs at 1, 3, and 5 years were 0.753, 0.791, and 0.737, respectively, for the TCGA dataset and 0.711, 0.700, and 0.673, respectively, for the GSE14520 dataset, suggesting that the performance of the risk signature is reliable.

In addition, the MCPcounter algorithm was used to assess the immune cell infiltration score based on the expression profile data of TCGA samples. The results showed six DICs between the high- and low-OSRG score groups, including CD8 T cells, cytotoxic lymphocytes, and natural killer (NK) cells. CD8^+^ T cells have been reported to play a vital role in protective immunity against intracellular pathogens and tumors (Ando et al., 2020). Stanton et al. found that tumor-infiltrating lymphocytes play vital roles in mediating chemotherapy response and improving the clinical outcomes of all breast cancer subtypes (Stanton and Disis, 2016). NK cells are powerful effectors of innate immunity and constitute the first line of defense against cancer (Guillerey, 2020). Moreover, GSEA results showed that the enrichment scores of the 16 immune gene sets were significantly different between the high- and low-OSRG score groups. In addition, eight HALLMARK gene set enrichment pathways, including fatty acid metabolism, glycolysis, and bile acid metabolism, showed significant differences between the two groups. Thus, these eight HALLMARK gene set enrichment pathways may be associated with liver cancer progression. Glycolysis is the key to supplying energy and producing metabolic end products to maintain the survival of tumor cells (Ganapathy-Kanniappan and Geschwind, 2013). Hu et al. (2017) revealed that phosphoglycerate kinase 1 (PGK1) is an important enzyme in the metabolic glycolytic pathway, and its acetylation enhances the proliferation and tumorigenesis of hepatocellular carcinoma cells. Feng et al. (2021) revealed that dysregulation of bile acid metabolism was associated with cancer cachexia. Thus, we speculated that these six DICs and eight HALLMARK gene enrichment pathways might be involved in liver cancer progression.

In addition, correlations between high and low OSRG scores and clinical information were evaluated, and the results showed that OSRG scores were significantly related to cluster, grade, pathological T, and stage. In addition, the stage and risk score were independent prognostic factors for clinical decision support in patients with lung adenocarcinoma. Furthermore, a nomogram was built using the stage and risk scores. Nomograms have become a popular tool for tumor prognosis owing to their intuitive visual performance and personalized application (Iasonos et al., 2008; Liu et al., 2019). Consistently, the nomogram in this study accurately estimated the survival probabilities of patients with liver cancer, and the performance of the nomogram was validated by measuring the C-index, calibration curves, and ROC curves. The results showed that the C-index of the nomogram was 0.703, and calibration plots revealed that the nomogram accurately estimated mortality. The nomogram ROC curve showed that the AUCs at 1, 3, and 5 years were 0.725, 0.751, and 0.720, respectively. Liu et al. (2020) identified a prognostic signature of epithelial ovarian cancer based on tumor immune microenvironment exploration, and the results showed that the AUCs of the nomogram were 0.70, 0.653, 0.723 for 1-year, 3-year, and 5-year OS, respectively, suggesting that the prognostic nomogram constructed in this study showed a good predictive ability and could accurately predict the survival of patients with liver cancer.

However, this study had numerous limitations. First, the data analyzed in this study were obtained from public databases, and external validation is needed. Second, the CIBERSORT algorithm was only used to evaluate the fractions of tumor-infiltrating immune cells, and other tools and flow cytometry are required to verify further the robustness of the results obtained in this study. Third, the seven OSRGs screened in this study should be tested in other cohorts and through further experimental analyses.

In summary, we constructed a reliable OSRG-related prognostic signature that is closely associated with the immune system, can accurately predict survival, and provides insights into predictive biomarkers or potential targets for patients with liver cancer.

## Data Availability

The original contributions presented in the study are included in the article/supplementary material, further inquiries can be directed to the corresponding author.

## References

[B1] Ando M. Ito M. Srirat T. Kondo T. Yoshimura A. (2020). Memory T cell, exhaustion, and tumor immunity. Immunol. Med. 43, 1–9. 10.1080/25785826.2019.1698261 31822213

[B2] Barrett T. Wilhite S. E. Ledoux P. Evangelista C. Kim I. F. Tomashevsky M. (2013). NCBI GEO: Archive for functional genomics data sets-update. Nucleic acids Res. 41, D991–D995. 10.1093/nar/gks1193 23193258 PMC3531084

[B3] Bhattacharya S. Andorf S. Gomes L. Dunn P. Schaefer H. Pontius J. (2014). ImmPort: Disseminating data to the public for the future of immunology. Immunol. Res. 58, 234–239. 10.1007/s12026-014-8516-1 24791905

[B4] Boland P. Wu J. (2018). Systemic therapy for hepatocellular carcinoma: Beyond sorafenib. Chin. Clin. Oncol. 7, 50. 10.21037/cco.2018.10.10 30395717

[B5] Cao W. Chen H. D. Yu Y. W. Li N. (2021). Changing profiles of cancer burden worldwide and in China: A secondary analysis of the global cancer statistics 2020. Chin. Med. J. Engl. 134, 783–791. 10.1097/CM9.0000000000001474 33734139 PMC8104205

[B6] Chen B. Khodadoust M. S. Liu C. L. Newman A. M. Alizadeh A. A. (2018). Profiling tumor infiltrating immune cells with CIBERSORT. Methods Mol. Biol. Clift. NJ) 1711, 243–259. 10.1007/978-1-4939-7493-1_12 PMC5895181 29344893

[B7] Chen Y. Liang Y. Chen Y. Ouyang S. Liu K. Yin W. (2021). Identification of prognostic metabolism-related genes in clear cell renal cell carcinoma. J. Oncol. 2021, 1–13. 10.1155/2021/2042114 PMC8490028 34616452

[B8] Chikara S. Nagaprashantha L. D. Singhal J. Horne D. Awasthi S. Singhal S. S. (2018). Oxidative stress and dietary phytochemicals: Role in cancer chemoprevention and treatment. Cancer Lett. 413, 122–134. 10.1016/j.canlet.2017.11.002 29113871

[B9] Feng L. Zhang W. Shen Q. Miao C. Chen L. Li Y. (2021). Bile acid metabolism dysregulation associates with cancer cachexia: Roles of liver and gut microbiome. J. cachexia, sarcopenia muscle 12, 1553–1569. 10.1002/jcsm.12798 34585527 PMC8718071

[B10] Ganapathy-Kanniappan S. Geschwind J. F. (2013). Tumor glycolysis as a target for cancer therapy: Progress and prospects. Mol. cancer 12, 152. 10.1186/1476-4598-12-152 24298908 PMC4223729

[B11] Gao X. Jiang S. Du Z. Ke A. Liang Q. Li X. (2019). KLF2 protects against osteoarthritis by repressing oxidative response through activation of Nrf2/ARE signaling *in vitro* and *in vivo* . Oxidative Med. Cell. Longev. 2019, 8564681. 10.1155/2019/8564681 PMC6885785 31827706

[B12] Guillerey C. (2020). NK cells in the tumor microenvironment. Adv. Exp. Med. Biol. 1273, 69–90. 10.1007/978-3-030-49270-0_4 33119876

[B13] Hinshaw D. C. Shevde L. A. (2019). The tumor microenvironment innately modulates cancer progression. Cancer Res. 79, 4557–4566. 10.1158/0008-5472.CAN-18-3962 31350295 PMC6744958

[B14] Hu H. Zhu W. Qin J. Chen M. Gong L. Li L. (2017). Acetylation of PGK1 promotes liver cancer cell proliferation and tumorigenesis. Hepatol. Baltim. Md) 65, 515–528. 10.1002/hep.28887 27774669

[B15] Huang H. (2018). Matrix metalloproteinase-9 (MMP-9) as a cancer biomarker and MMP-9 biosensors: Recent advances. Sensors (Basel, Switz., 18(10):3249. 10.3390/s18103249 PMC6211011 30262739

[B16] Iasonos A. Schrag D. Raj G. V. Panageas K. S. (2008). How to build and interpret a nomogram for cancer prognosis. J. Clin. Oncol. official J. Am. Soc. Clin. Oncol. 26, 1364–1370. 10.1200/JCO.2007.12.9791 18323559

[B17] Jin Y. Sun Z. Geng J. Yang L. Song Z. Song H. (2019). IL-21 reinvigorates exhausted natural killer cells in patients with HBV-associated hepatocellular carcinoma in STAT1-depedent pathway. Int. Immunopharmacol. 70, 1–8. 10.1016/j.intimp.2019.02.007 30780004

[B18] Kim J. Kim H. Lee J. Choi I. J. Kim Y. I. (2020). Antioxidant-rich diet, GSTP1 rs1871042 polymorphism, and gastric cancer risk in a hospital-based case-control study. Front. Oncol. 10, 596355. 10.3389/fonc.2020.596355 33634021 PMC7902036

[B19] Klaunig J. E. (2018). Oxidative stress and cancer. Curr. Pharm. Des. 24, 4771–4778. 10.2174/1381612825666190215121712 30767733

[B20] Kudryavtseva A. V. Krasnov G. S. Dmitriev A. A. Alekseev B. Y. Kardymon O. L. Sadritdinova A. F. (2016). Mitochondrial dysfunction and oxidative stress in aging and cancer. Oncotarget 7, 44879–44905. 10.18632/oncotarget.9821 27270647 PMC5216692

[B21] Langfelder P. Horvath S. (2008). Wgcna: an R package for weighted correlation network analysis. BMC Bioinforma. 9, 559. 10.1186/1471-2105-9-559 PMC2631488 19114008

[B22] Lin P. H. Aronson W. Freedland S. J. (2019). An update of research evidence on nutrition and prostate cancer. Urol. Oncol. 37, 387–401. 10.1016/j.urolonc.2017.10.006 29103966

[B23] Liu J. Meng H. Nie S. Sun Y. Jiang P. Li S. (2020). Identification of a prognostic signature of epithelial ovarian cancer based on tumor immune microenvironment exploration. Genomics 112, 4827–4841. 10.1016/j.ygeno.2020.08.027 32890701

[B24] Liu Y. Wen Q. J. Yin Y. Lu X. T. Pu S. H. Tian H. P. (1990). FASLG polymorphism is associated with cancer risk. Eur. J. cancer. 45, 2574–2578. 10.1016/j.ejca.2009.04.001 19403301

[B25] Liu Y. Wu L. Ao H. Zhao M. Leng X. Liu M. (2019). Prognostic implications of autophagy-associated gene signatures in non-small cell lung cancer. Aging 11, 11440–11462. 10.18632/aging.102544 31811814 PMC6932887

[B26] Lu Y. Qin H. Jiang B. Lu W. Hao J. Cao W. (2021). KLF2 inhibits cancer cell migration and invasion by regulating ferroptosis through GPX4 in clear cell renal cell carcinoma. Cancer Lett. 522, 1–13. 10.1016/j.canlet.2021.09.014 34520818

[B27] Lv Z. Li W. Wei X. (2020). S100A9 promotes prostate cancer cell invasion by activating TLR4/NF-κB/integrin β1/FAK signaling. OncoTargets Ther. 13, 6443–6452. 10.2147/OTT.S192250 PMC7435298 32884282

[B28] Marengo A. Rosso C. Bugianesi E. (2016). Liver cancer: Connections with obesity, fatty liver, and cirrhosis. Annu. Rev. Med. 67, 103–117. 10.1146/annurev-med-090514-013832 26473416

[B29] Numakawa T. Odaka H. Adachi N. (2018). Actions of brain-derived neurotrophin factor in the neurogenesis and neuronal function, and its involvement in the pathophysiology of brain diseases. Int. J. Mol. Sci. 19, 3650. 10.3390/ijms19113650 30463271 PMC6274766

[B30] Olingy C. E. Dinh H. Q. Hedrick C. C. (2019). Monocyte heterogeneity and functions in cancer. J. Leukoc. Biol. 106, 309–322. 10.1002/JLB.4RI0818-311R 30776148 PMC6658332

[B31] Oyama Y. Nagao S. Na L. Yanai K. Umebayashi M. Nakamura K. (2021). TrkB/BDNF signaling could Be a new therapeutic target for pancreatic cancer. Anticancer Res. 41, 4047–4052. 10.21873/anticanres.15205 34281873

[B32] Pucci M. Raimondo S. Urzì O. Moschetti M. Di Bella M. A. Conigliaro A. (2021). Tumor-derived small extracellular vesicles induce pro-inflammatory cytokine expression and PD-L1 regulation in M0 macrophages via IL-6/STAT3 and TLR4 signaling pathways. Int. J. Mol. Sci. 22, 12118. 10.3390/ijms222212118 34829995 PMC8621495

[B33] Reimand J. Isserlin R. Voisin V. Kucera M. Tannus-Lopes C. Rostamianfar A. (2019). Pathway enrichment analysis and visualization of omics data using g:Profiler, GSEA, Cytoscape and EnrichmentMap. Nat. Protoc. 14, 482–517. 10.1038/s41596-018-0103-9 30664679 PMC6607905

[B34] Rizvi A. A. Karaesmen E. Morgan M. Preus L. Wang J. Sovic M. (2019). gwasurvivr: an R package for genome-wide survival analysis. Bioinforma. Oxf. Engl. 35, 1968–1970. 10.1093/bioinformatics/bty920 PMC7963072 30395168

[B35] Saheb Sharif-Askari N. Saheb Sharif-Askari F. Mdkhana B. Hussain Alsayed H. A. Alsafar H. Alrais Z. F. (2021). Upregulation of oxidative stress gene markers during SARS-COV-2 viral infection. Free Radic. Biol. Med. 172, 688–698. 10.1016/j.freeradbiomed.2021.06.018 34186206 PMC8233550

[B36] Sajadimajd S. Khazaei M. (2018). Oxidative stress and cancer: The role of Nrf2. Curr. cancer drug targets 18, 538–557. 10.2174/1568009617666171002144228 28969555

[B37] Sakaguchi S. Mikami N. Wing J. B. Tanaka A. Ichiyama K. Ohkura N. (2020). Regulatory T cells and human disease. Annu. Rev. Immunol. 38, 541–566. 10.1146/annurev-immunol-042718-041717 32017635

[B38] Shi J. Jiang D. Yang S. Zhang X. Wang J. Liu Y. (2020). LPAR1, correlated with immune infiltrates, is a potential prognostic biomarker in prostate cancer. Front. Oncol. 10, 846. 10.3389/fonc.2020.00846 32656075 PMC7325998

[B39] Sia D. Villanueva A. Friedman S. L. Llovet J. M. (2017). Liver cancer cell of origin, molecular class, and effects on patient prognosis. Gastroenterology 152, 745–761. 10.1053/j.gastro.2016.11.048 28043904 PMC12160040

[B40] Soysal S. D. Tzankov A. Muenst S. E. (2015). Role of the tumor microenvironment in breast cancer. Pathobiology J. Immunopathol. Mol. Cell. Biol. 82, 142–152. 10.1159/000430499 26330355

[B41] Stanton S. E. Disis M. L. (2016). Clinical significance of tumor-infiltrating lymphocytes in breast cancer. J. Immunother. cancer 4, 59. 10.1186/s40425-016-0165-6 27777769 PMC5067916

[B42] Sung H. Ferlay J. Siegel R. L. Laversanne M. Soerjomataram I. Jemal A. (2021). Global cancer statistics 2020: GLOBOCAN estimates of incidence and mortality worldwide for 36 cancers in 185 countries. CA a cancer J. Clin. 71, 209–249. 10.3322/caac.21660 33538338

[B43] Tajbakhsh A. Mokhtari-Zaer A. Rezaee M. Afzaljavan F. Rivandi M. Hassanian S. M. (2017). Therapeutic potentials of BDNF/TrkB in breast cancer; current status and perspectives. J. Cell. Biochem. 118, 2502–2515. 10.1002/jcb.25943 28230291

[B44] Wang C. Liao Y. He W. Zhang H. Zuo D. Liu W. (2021a). Elafin promotes tumour metastasis and attenuates the anti-metastatic effects of erlotinib via binding to EGFR in hepatocellular carcinoma. J. Exp. Clin. cancer Res. CR 40, 113. 10.1186/s13046-021-01904-y 33771199 PMC7995733

[B45] Wang G. Duan P. Liu F. Wei Z. (2021b). Long non-coding RNA CASC7 suppresses malignant behaviors of breast cancer by regulating miR-21-5p/FASLG axis. Bioengineered 12, 11555–11566. 10.1080/21655979.2021.2010372 34889164 PMC8809951

[B46] Wang Q. Gao X. Yuan Z. Wang Z. Meng Y. Cao Y. (2014). Methionine enkephalin (MENK) improves lymphocyte subpopulations in human peripheral blood of 50 cancer patients by inhibiting regulatory T cells (Tregs). Hum. vaccines Immunother. 10, 1836–1840. 10.4161/hv.28804 PMC4186042 25424790

[B47] Wilkerson M. D. Hayes D. N. (2010). ConsensusClusterPlus: A class discovery tool with confidence assessments and item tracking. Bioinforma. Oxf. Engl. 26, 1572–1573. 10.1093/bioinformatics/btq170 PMC2881355 20427518

[B48] Yoshihara K. Shahmoradgoli M. Martínez E. Vegesna R. Kim H. Torres-Garcia W. (2013). Inferring tumour purity and stromal and immune cell admixture from expression data. Nat. Commun. 4, 2612. 10.1038/ncomms3612 24113773 PMC3826632

[B49] Yu Y. Zheng S. Zhang S. Jin W. Liu H. JinM. (2014). Polymorphisms of inflammation-related genes and colorectal cancer risk: A population-based case-control study in China. Int. J. immunogenetics 41, 289–297. 10.1111/iji.12119 24762198

[B50] Zhang S. Tong Y. X. Zhang X. H. Zhang Y. J. Xu X. S. Xiao A. T. (2019). A novel and validated nomogram to predict overall survival for gastric neuroendocrine neoplasms. J. Cancer 10, 5944–5954. 10.7150/jca.35785 31762804 PMC6856574

